# Transcriptome profile of the human placenta

**DOI:** 10.1007/s10142-017-0555-y

**Published:** 2017-03-01

**Authors:** Marta Majewska, Aleksandra Lipka, Lukasz Paukszto, Jan Pawel Jastrzebski, Kamil Myszczynski, Marek Gowkielewicz, Marcin Jozwik, Mariusz Krzysztof Majewski

**Affiliations:** 1 Department of Human Physiology, Faculty of Medical Sciences, University of Warmia and Mazury in Olsztyn, Warszawska Str 30, 10-082 Olsztyn, Poland; 2 Department of Gynecology and Obstetrics, Faculty of Medical Sciences, University of Warmia and Mazury in Olsztyn, Niepodleglosci Str 44, 10-045 Olsztyn, Poland; 3 Department of Plant Physiology, Genetics and Biotechnology, Faculty of Biology and Biotechnology, University of Warmia and Mazury in Olsztyn, Oczapowskiego Str 1A, 10-719 Olsztyn-Kortowo, Poland

**Keywords:** RNA-seq, Human, Placenta, Transcriptome

## Abstract

The human placenta is a particular organ that inseparably binds the mother and the fetus. The proper development and survival of the conceptus relies on the essential interplay between maternal and fetal factors involved in cooperation within the placenta. In our study, high-throughput sequencing (RNA-seq) was applied to analyze the global transcriptome of the human placenta during uncomplicated pregnancies. The RNA-seq was utilized to identify the global pattern of the gene expression in placentas (*N* = 4) from women in single and twin pregnancies. During analyses, we obtained 228,044 transcripts. More than 91% of them were multi-exon, and among them 134 were potentially unknown protein coding genes. Expression levels (FPKM) were estimated for 38,948 transcriptional active regions, and more than 3000 of genes were expressed with FPKM >20 in each sample. Additionally, all unannotated transcripts with estimated FPKM values were localized on the human genome. Highly covered splice junctions unannotated in the human genome (6497) were identified, and among them 30 were novel. To gain a better understanding of the biological implications, the assembled transcripts were annotated with gene ontology (GO) terms. Single nucleotide variants were predicted for the transcripts assigned to each analyzed GO category. Our results may be useful for establishing a general pattern of the gene expression in the human placenta. Characterizing placental transcriptome, which is crucial for a pregnancy’s outcome, can serve as a basis for identifying the mechanisms underlying physiological pregnancy, as well as may be useful for an early detection of the genomic defects.

**Electronic supplementary material:**

The online version of this article (doi:10.1007/s10142-017-0555-y) contains supplementary material, which is available to authorized users.

## Introduction

Next-generation sequencing (NGS) applied to the transcriptome (RNA-Seq) is a technology that uncovers broad amounts of novel information about the transcript (Lerch et al. 2012). RNA-Seq is a powerful tool suitable for a wide range of large-scale tissue-specific profiling, which includes analyses of transcriptome simultaneously for mutation, structural alteration, and expression, even the reconstruction of the entire transcriptome in the absence of a reference genome (Hui 2014). Despite the need for advanced bioinformatic and time-consuming data analysis, the RNA-seq still provides a strong alternative to the use of microarrays in gene expression studies (Grada and Weinbrecht 2013). The discovery of specific genes crucial for placental development and function can help in understanding and identifying the mechanisms underlying both normal and pathological pregnancies. Detailing the placental tissue transcriptome could provide a valuable resource for genomic studies related to placental disease.

Particularly in the context of established basis, the completion of the human genome sequencing project and the accessibility of the reference human genome enabled the application of genomic knowledge in clinical practice. The current progress of NGS technology is vertiginous, and genomic medicine driven by NGS may improve the diagnosis, prognosis, and therapy of many to-date incurable diseases (Ley et al. 2008; Dietz 2010).

Reproductive success is deeply linked with precise maternal-embryo communication, which results in correct placental functioning. The placenta is a primary organ essential for maintaining the proper development of the human fetus during pregnancy. Any disorder in this unique and delicate molecular crosstalk will have implications resulting in restricted progress in successful and proper embryo growth. Disturbances, if more severe, may lead to pregnancy failure, or even influence the developmental potential of offspring to adulthood (Fazeli and Pewsey 2008; Fan et al. 2016). Besides its hormonal and nutritive functions, the placenta also serves as a barrier against the maternal immune system, the gas exchange compartment, as well as the fetal waste disposal organ.

The nutritional status of the mother and infant from conception until early childhood, as well as maternal prenatal distress and compromise affect the early programming of the immune function, which might, in consequence, influence the neuro-development of the embryo (Marques et al. 2013).

Moreover, hormones produced by the placenta adjust maternal physiology to couple the body changes during the pregnancy’s progress (Cross et al. 2003; Watson and Cross 2005). Additionally, significant differences in efficiency and growth, depending on the sex of the fetus, have been observed (Forsén et al. 1999; Eriksson et al. 2010). Differences in placental gene expression may also be correlated with the rate of growth and body length or weight as observed in males at birth, in comparison with females with equivalent placental size (Misra et al. 2009).

The properly functioning placenta depends on the finely tuned regulation of relevant genes that affect its structural integrity, and the proper growth and development of its structural components. Thus, any disturbances in gene expression may represent a major molecular mechanism underlying pathological pregnancies (Mikheev et al. 2008).

A profound penetration of the spatial and temporal changes of the placental transcriptome during development and pathology of the pregnancy should be considered essential in identifying the origins of diseases such as preeclampsia (PE) and intrauterine growth restriction (IUGR) that cause stillbirth or preterm birth. The consequences may extend into adult life, with a metabolic syndrome being a dramatic example. The advanced RNA-seq is an effective tool for analysis of the placental transcriptome to elucidate possible molecular mechanisms leading to these diseased states, and to identify candidate biomarkers (Cox et al. 2015).

The frequency of multiple gestations has increased over the last few decades, mainly due to the use of assisted reproductive technologies with the increased maternal age at childbearing. Twin pregnancies are at higher perinatal risk for both mothers and fetuses than singleton pregnancies (Chauhan et al. 2010; Hubinont et al. 2015).

The aim of the present study was to identify the transcriptome landscape of the human placenta during uncomplicated single and twin pregnancies to establish the pattern of normal placental gene expression for further comprehensive analyses.

## Materials and methods

### Collection of placental samples and ethics statement

All clinical samples were collected at the Clinical Ward for Gynecology, Obstetrics and Oncological Gynecology at the Regional Specialist Hospital in Olsztyn following informed written consent from mothers. The experimental protocol was approved by the Bioethics Committee of the Warmia-Mazury Medical Chamber (OIL.164/15/Bioet) in Olsztyn, Poland.

### Placental tissue collection

Placentas (*N* = 4) were collected from healthy women after uncomplicated pregnancy from term pregnancy (Table 1), who underwent a scheduled Cesarean section before the onset of labor. Samples of placental tissues were collected immediately after delivery; then, small pieces of placentas were frozen in liquid nitrogen. Preserved tissues were stored in the Laboratory of Molecular Diagnostics at −70 °C until further analyses.

**Table 1:** Characteristics of collected placental samples used in RNA-seq

Sample code	Maternal age (years)	Gestational age (weeks)	Sex of fetus	Multiplicity of gestation	Birth weight (g)
Hs_p3	34	41	♂	Single	3900
Hs_p9	32	36	♂	Twin	2870
Hs_p12	33	37	♀	Twin	2150
Hs_p14	36	39	♀	Single	2720

### Total RNA extraction and purification

RNA was isolated from the placental tissues using the Qiagen RNeasy Kit according to the manufacturer’s recommendations. The RNase-Free DNase Set (Qiagen) was used to obtain high-quality RNA with DNA-ase digestion. RNA integrity (purity and concentration) was evaluated using the Tecan InfiniteM200 (Tecan Group AG). The RNA integrity number (RIN) was measured on a Bioanalyzer 2100 (Agilent). Only samples with the RIN >8 were subjected to further analysis. Fifteen microliters of total RNA extracted from each sample was delivered to OpenExome (Poland) in order to prepare a cDNA library and perform RNA-seq.

### cDNA library construction and transcriptome sequencing

Double-stranded cDNA libraries were prepared from total extracted RNA using the TruSeq Stranded mRNA LT Sample Prep Kit v3 (Illumina) following manufacturer’s instructions. The concentration of extracted RNA ranged from 10.07 to 24.06 ng/μl. Briefly, RNA samples were first purified with two oligo-dT selection (poly(A) enrichment using oligo-dT beds), and then fragmented and reverse transcribed into double-stranded complementary DNA. The resulting cDNA libraries of each sample were tagged with an indexed adapter prior to sequencing. The indexed libraries were diluted and pooled in equimolar ratios, and then the libraries were pair-end sequenced (101 cycles for read 1, 7 cycles for the index read, and 101 cycles for read 2) on the HiSeq2500 (Illumina), 2 × 100 bp reads were obtained.

### Quality control and mapping to reference

The FastQC software (www.bioinformatics.babraham.ac.uk) was utilized for the evaluation of the quality control of the sequencing process. In order to remove Illumina adaptors and poly(A) stretches out of raw sequencing reads, the Trimmomatic tool (Bioinformatics, btu170) was used. Also, reads shorter than 50 bp or quality lower than Phred < 20 were removed from the dataset. Trimmed sequences were aligned to the human reference genome (Homo_sapiens.GRCh38.dna.primary_assembly.fa) with annotation (Homo_sapiens.GRCh38.80.gtf) file. Mapping was performed with the STAR (ver. 2.4) mapper with the following parameters: --outFilterType BySJout --outFilterMultimapNmax 20 --alignSJoverhangMin 8 --alignSJDBoverhangMin 1 --outFilterMismatchNmax 999 --outFilterMismatchNoverLmax 0.04 --alignIntronMin 20 --alignIntronMax 1,000,000 --alignMatesGapMax 1,000,000. As a result, we obtained alignment of the trimmed reads to the reference genome (BAM files). The number of reads mapped to: exonic, intronic, UTRs, or intergenic regions was quantified using the CollectRnaSeqMetrics tool in the Picard 2.1.1 software (http://picard.sourceforge.net).

### Transcriptional active regions

Clean reads were mapped on the human genome and as a result transcriptional active regions (TARs) were obtained. If TAR had an annotation (meaning assigned gene, non-coding region, processed transcript, miRNA, pseudogene, etc.), it was classified as annotated, if it did not have any annotation it was classified as unannotated.

Cufflinks and StringTie tools (Trapnell et al. 2012; Pertea et al. 2015) were used to estimate expression values from BAM files, for both annotated and unannotated TARs (expressed in at least one of four samples). All TARs in each sample were divided due to expression levels: FPKM <1, 1 ≤ FPKM <10, 10 ≤ FPKM <20 or FPKM ≥ 20. Additionally, unannotated regions were verified by searching for identical transcripts in non-redundant protein NCBI (using BLASTx-fast) and Ensembl (using BLASTx) databases. Hits selected after BLASTx-fast verification were grouped into four categories: (1) known human genes (similarity >90%); (2) unknown one exon genes (identity <90% and ORF >300 bp, number of exon = 1); (3) unknown multiple exon genes (identity <90% and ORF >300 bp, number of exon >1); and (4) non-coding transcripts (identity <90% or non-hits targets and ORF <300 bp). Additionally, all unannotated transcripts (for four samples) with estimated FPKM values were localized in the human genome. Obtained results were presented in Circos (Krzywinski et al. 2009).

Additionally, to specify the above analysis for each sample, cut-off FPKM (equal to 0.25) was applied to remove unannotated transcripts with low expression levels. Finally, 5320 TARs were expressed in more than one sample, 2742 TARs were expressed in more than two examined samples, and 880 unannotated TARs were expressed in all examined samples.

### Alternative splicing events

Among all splice junctions (SJ.out.tab files) generated by STAR, novel exon-exon junction sites were selected. The number of uniquely mapped reads crossing the junction was set for 5×. RefGenome (bioconductor library in R) was used to overlap selected junctions with transcripts. Overlapped transcripts were merged with potential novel isoforms generated by Cufflinks-StringTie analyses. BLASTx was used to verify potential splice isoforms and indicate novel ones.

### Gene ontology classification

The StringTie software was also used to generate additional annotations on the basis of mapped reads. Genes with FPKM >1 (sum of each sample) were selected, and the longest isoform of each transcript (unigene) was assigned to Gene Ontology (GO) terms in BLAST2GO software with BLASTx-fast; *p* value = 10e-5, human reference (Conesa et al. 2005). For those selected transcripts, InterproScan was applied to the predicted protein signature database including Pfam, Panther, and SMART (Hunter et al. 2009). Among the processes involved in placental development, five GO terms were selected: embryo development, placenta development, embryo implantation, maternal placenta development, and embryo placenta development. For the genes from selected GO terms, alternative splicing sites were identified and counted (Geneious and StringTie). Single nucleotide variants (SNV) were predicted by GATK (McKenna et al. 2010) and the Picard tool.

## Results

### Statistics of placenta transcriptome profile

The RNA-seq of the constructed cDNA libraries allowed the characterization of the transcriptome profile of the human placenta. High-throughput sequencing on an Illumina platform generated 2 × 119,560,140 total raw paired-end reads. After trimming, 2 × 109,363,183 reads with good quality were obtained. Among all clean reads, 86.76, 85.76, 84.29, and 87.72% were uniquely mapped for each sample, respectively. Moreover, ~8.71% were mapped to multiple loci (2–20), ~0.0125% to too many loci (>20), and only ~0.0325% of reads were unmapped (Table 2).

**Table 2:** Overall statistics of human placenta transcriptome profile for each sample (for definitions of samples see Table 1)

Sample	Hs_p3	Hs_p9	Hs_p12	Hs_p14
Number of raw paired-end reads	44,777,519	26,493,951	20,537,685	27,750,985
Number of trimmed paired-end reads	41,246,951	24,145,451	18,638,013	25,332,768
Average input read length (bp)	196	196	197	197
Unique reads:				
Uniquely mapped reads number	35,787,077	20,706,449	15,709,994	22,220,706
Uniquely mapped reads (%)	86.76	85.76	84.29	87.72
Mapped length	196	197	197	197
Number of splices: total	23,163,924	11,420,872	10,028,766	12,182,247
Number of splices: annotated	22,933,481	11,300,279	9,909,568	12,046,860
Number of splices: GT/AG	23,007,429	11,340,273	9,975,740	12,099,453
Number of splices: GC/AG	141,744	72,166	48,097	74,841
Number of splices: AT/AC	14,751	8433	4929	7953
Mismatch rate per base (%)	0.17	0.12	0.14	0.11
Deletion rate per base (%)	0.01	0.01	0.01	0.01
Deletion average length	1.85	1.87	1.91	1.88
Insertion rate per base %)	0.01	0.01	0.01	0.01
Insertion average length	1.40	1.34	1.35	1.31
Multi-mapping reads:				
Number of reads mapped to multiple loci	4,824,177	1,760,204	1,538,463	1,924,603
Reads mapped to multiple loci (%)	11.70	7.29	8.25	7.60
Number of reads mapped to too many loci	3703	3568	2696	4788
Reads mapped to too many loci (%)	0.01	0.01	0.01	0.02
Unmapped reads:				
Reads unmapped: too many mismatches (%)	0.03	0.02	0.03	0.02
Reads unmapped: too short (%)	1.48	6.89	7.38	4.61
Reads unmapped: other (%)	0.02	0.04	0.03	0.04

*Hs* human sample

**Fig. 1:**
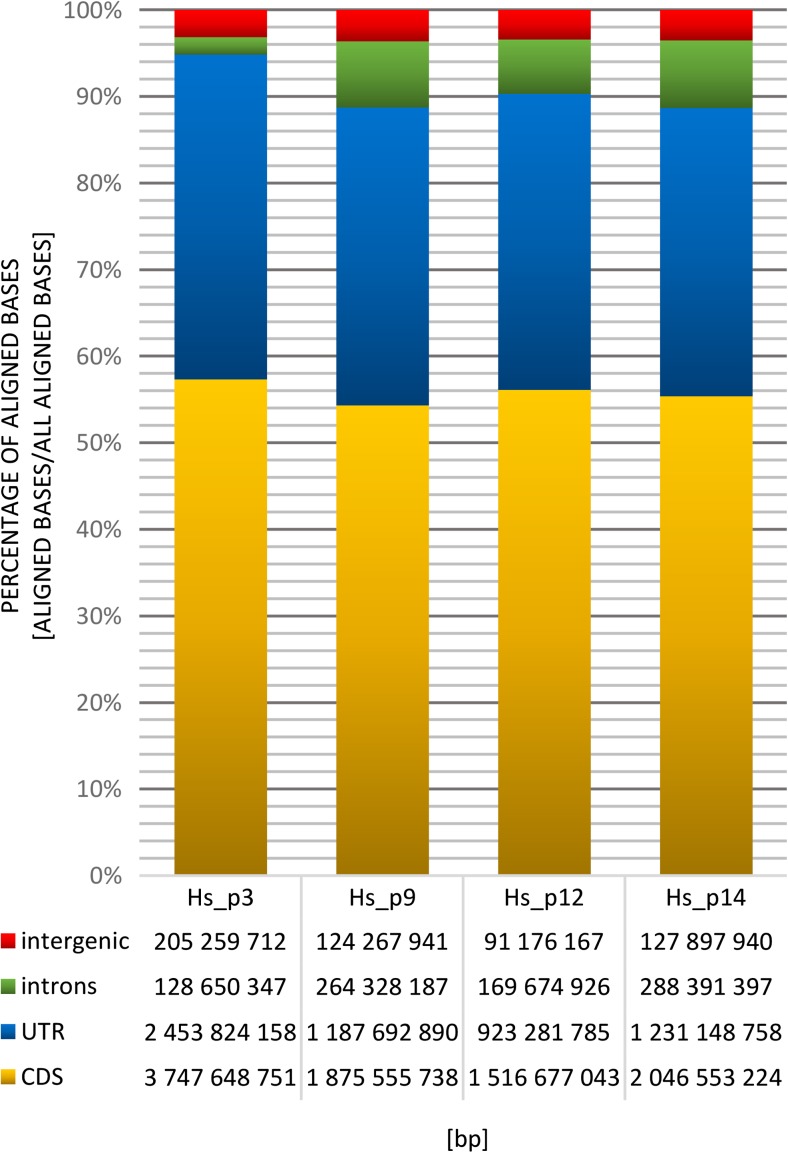
Aligned bases in human genome. Percentage (%) and distribution (bp) of bases mapped to the human genome within intergenic, intronic, UTR, and coding regions

For all input samples, the percentage distribution of aligned bases is mostly very similar: ~3.4% of aligned bases were derived from intergenic regions, ~34.85% of bases originated from UTR, and ~55.8% from coding regions (Fig. 1). Significant discrepancies in aligned bases distribution were detected within intronic regions, between one and other three samples, 2 and ~7.3% respectively (Fig. 1).

### Transcriptional active regions

**Fig. 2:**
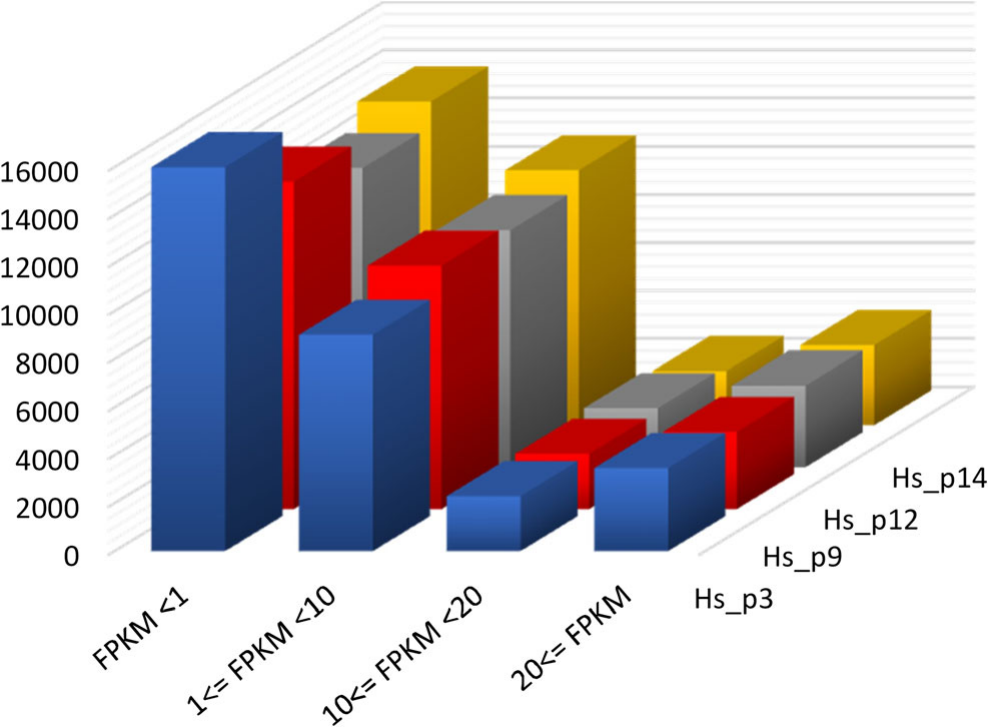
Overview of the transcripts’ expression. The number of mapped regions assigned to different ranges of FPKM in each sample. The FPKM value of each gene was calculated by CUFFLINKS

Cufflinks and StringTie generated 228,044 total transcripts, with more than 91% of them being multi-exon transcripts (Table 3). The uniquely mapped reads were used to estimate the expression levels (FPKM) of 38,948 TARs (annotated and unannotated). To overview the gene expression, FPKM values were divided into appropriate intervals. Based on the FPKM value, more than 3000 of genes were expressed with FPKM >20 in each sample. The largest number of genes was expressed below 1 FPKM. The general pattern of the gene expression was similar in all samples (Fig. 2).

**Table 3:** Overview of transcriptome assembly in human placental tissues

Total transcript	228,044
Multi-exon transcript	208,639
Total TARs	38,948
Multi-exon TARs	23,388
Annotated TARs	29,538
Unannotated TARs (not overlapping with the annotation)	9,434
Potentially novel isoform	44,569
Isoforms per TAR	5.86

*TAR* transcriptional active regions

Among identified TARs, 29,514 were overlapping with the annotated transcripts from the NCBI RefSeq and Ensembl database. The remaining 9434 TARs were unannotated. The obtained RNA-seq data also enabled the prediction of 44,569 potentially novel isoforms, which on average is 5.86 isoforms per TAR (Table 3).

**Fig. 3:**
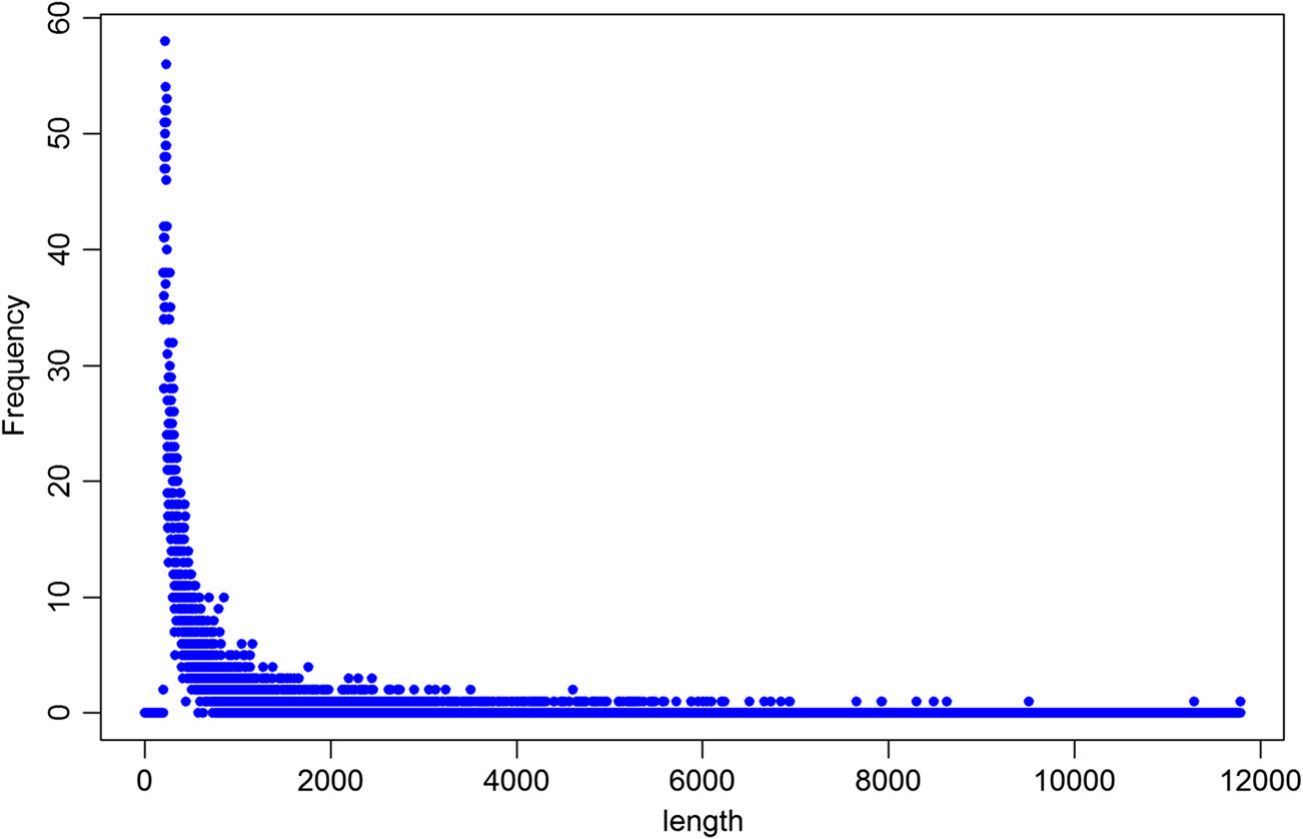
Distribution of non-coding RNA according to length (bp) in the human placenta

Based on a search against the human non-redundant (nr) database, unannotated regions were divided into four different groups of transcripts: over 90% similarity with nr database (558 transcripts), less than 90% similarity with ORF longer than 300 bp composed of less than 2 exons (754 transcripts), less than 90% similarity with ORF longer than 300 bp composed of at least 2 exons (138 transcripts), and less than 90% similarity with ORF shorter than 300 bp (8219 transcripts). Additionally, the last group, classified as non-coding RNA, was analyzed according to the length of sequences. We revealed 639.48 bp as the average length of transcripts longer than 200 bp, 2931 transcripts were long non-coding RNA (>500 bp), and the longest transcript was 11,781 bp in length (Fig. 3).

**Fig. 4:**
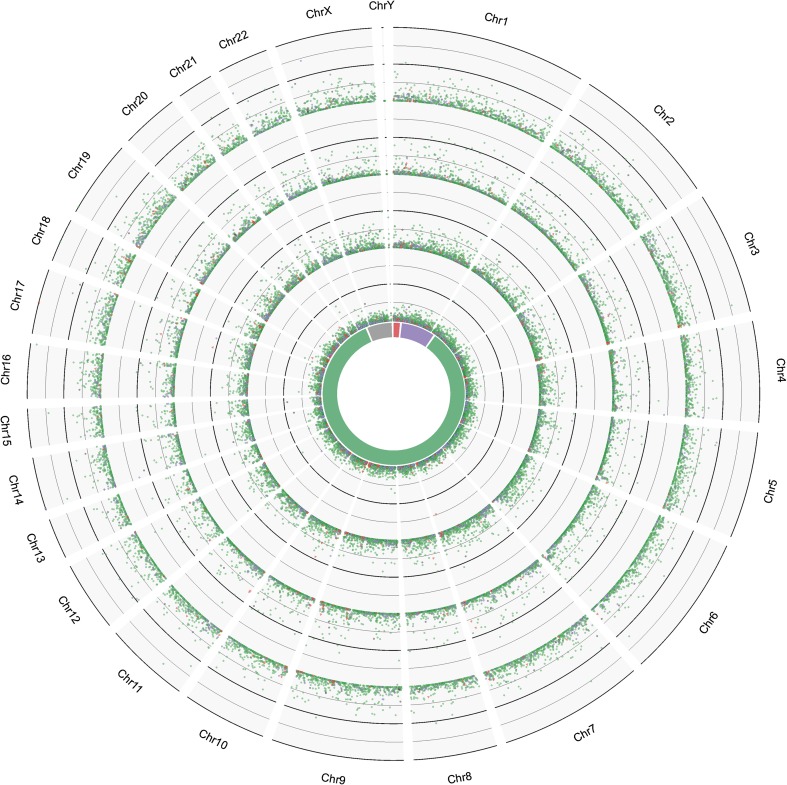
Comparison of unannotated transcripts’ distribution and expression level across the human genome. Four outer tracks depict the gene expression level (FPKM ranges from 0 to 20) of each placental sample. The most outer - Hs_p14 followed by Hs_p12, Hs_p9, and Hs_p3. *Colored tags* represent four different groups of transcripts: >90% similarity with the Human non-redundant database (*gray circles*), <90% similarity with ORF <300 bp (*green circles*), <90% similarity with ORF >300 bp composed of <2 exons (*purple circles*), <90% similarity with ORF >300 bp composed of >2 exons (*red triangles*). The *innermost circle* shows the percentage of every group to the total number of unannotated transcripts (9669)

All of the unannotated transcripts with estimated FPKM values were localized on the human genome (Fig. 4).

### Alternative splicing events

**Fig. 5:**
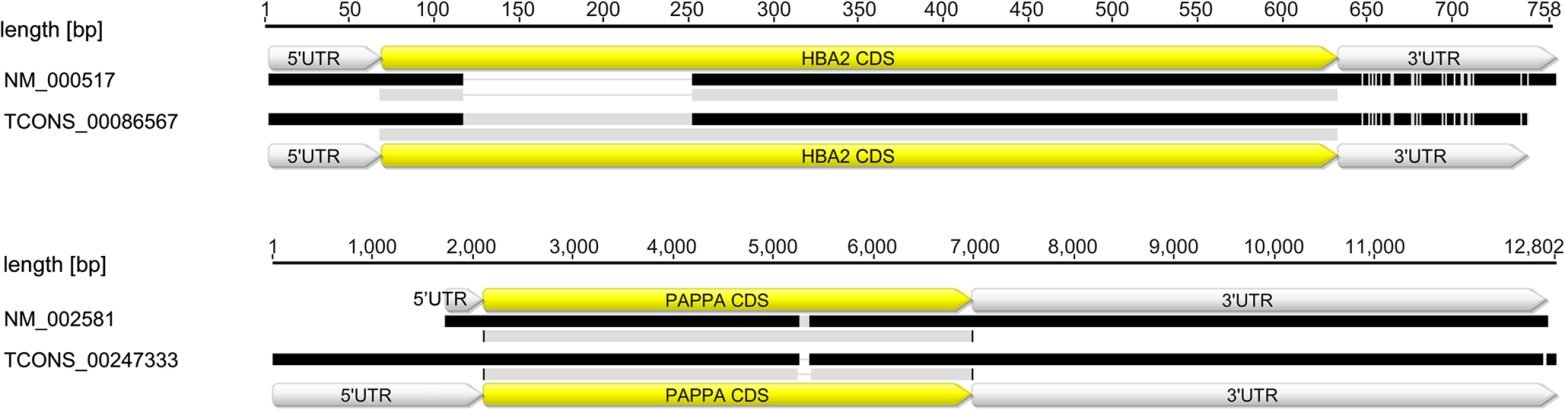
Pairwise alignment of examples of differential splicing events identified in *HBA2* and *PAPPA*. The 5′ untranslated region (UTR), coding DNA sequence (CDS), 3′ UTR are indicated

STAR generated 239,458, 219,385, 235,672, and 281,038 splice junction sites separately for each sample, Hs_p14, Hs_p12, Hs_p9, and Hs_p3, respectively. Among all splice junctions, 6497 fulfilled the following conditions: novel exon-exon junction and uniquely mapped reads crossing the junction >5×. Overlapping with reference transcripts coordinates and merging with potential novel isoforms (StringTie) generated 2766 contigs. Among this group, BLASTx and non-redundant NCBI GenPept database confirmed 30 as novel splice junctions. Among them, pregnancy-associated plasma protein A (*PAPPA*) and hemoglobin subunit alpha (*HBA*) were chosen to broaden the analysis, namely, mapping and comparison with the human reference transcriptome and genome (Fig. 5).

### Functional annotation based on GO categorization and KEGG annotation

To gain a better understanding of the biological implications, the assembled transcripts were annotated with gene ontology (GO) terms, based on the “best hit” BLASTx search in the Blast2GO pipeline. Unigenes with sum of FPKM >1 in all samples and FPKM >0.25 (in at least 3 samples) were annotated to three main GO categories: biological processes (82,751), cellular components (33,483), and molecular functions (32,625). Additionally, InterproScan predicted 3306 signatures in Panther, 16,994 in Pfam and 9145 in the SMART database (Online Resource 1). The majority of unigenes were assigned to biological processes GO category (51.3%), distributed in 27 subcategories, followed by cellular components (37%) and molecular functions (11.7%), with 19 and 17 subcategories, respectively.

The most overrepresented GO terms in biological processes were the cellular process (10,123) and single-organism process (8431). In the cellular component category, many transcripts were associated with intracellular membrane-bounded organelle (7424) and cytoplasm (7533). The molecular function category was mostly dominated by protein binding (7419), ion binding (4305), and organic cyclic compound binding (4020).

**Fig. 6:**
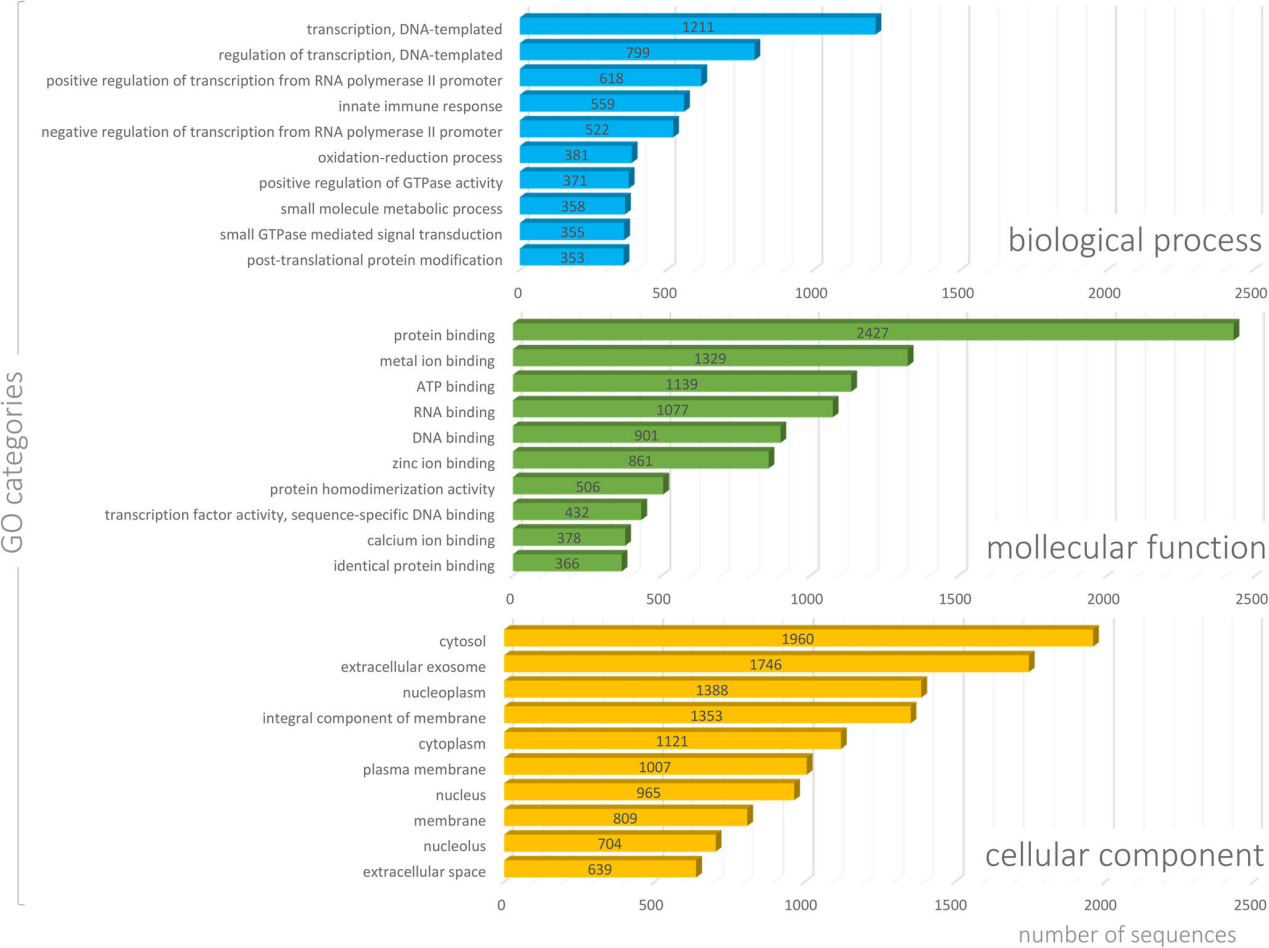
GO (Gene Ontology) classification of the unigenes. The results of the top 10 GO terms are presented for three main categories:biological process (*blue bars*), molecular function (*green bars*), and cellular component (*yellow bars*). Values in bars indicate the number of genes assigned to each process

**Fig. 7:**
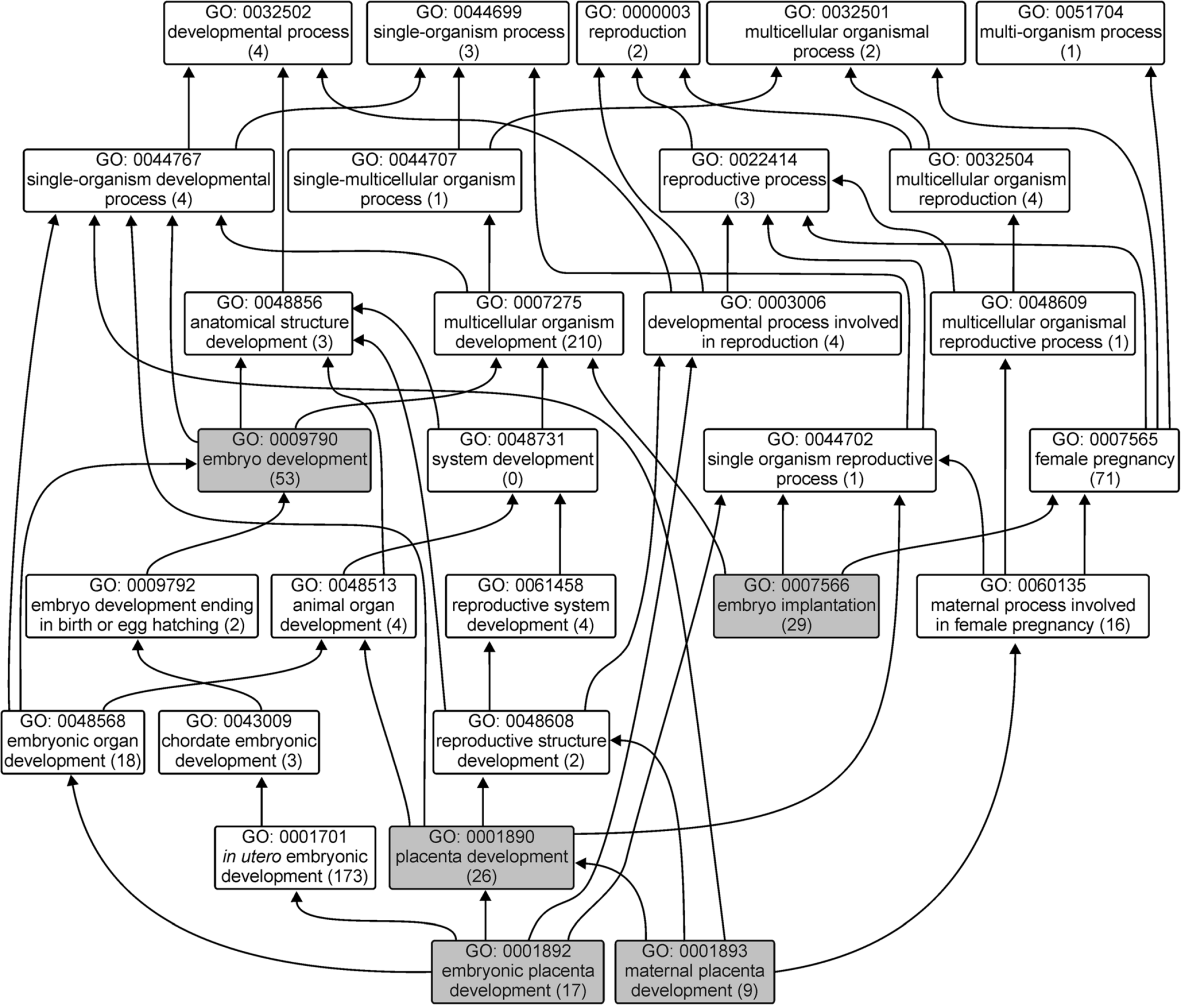
Distribution of human genes (within biological processes category) involved in five main reproduction processes (embryo development, embryo implantation, placenta development, embryonic placenta development and maternal placenta development). The numbers in parentheses indicate the number of genes assigned to each GO (Gene Ontology) process

The top 10 represented GO terms (Fig. 6a and Online Resource 2) for the biological process were transcription (1211), regulation of transcription (799), and positive regulation of transcription from RNA polymerase II promoter (618). For molecular function, the top represented terms (Fig. 6b and Online Resource 2) were protein binding (2427), metal ion binding (1329), and ATP binding (1139). Lastly, the top cellular component GO terms (Fig. 6c and Online Resource 2) were cytosol (1960), extracellular exosome (1746), and nucleoplasm (1388). Due to our concern about widely understood reproduction, we choose to present GO categories associated with this process. Thorough GO analyses revealed that 53 genes were assigned to the embryo development category. Furthermore, 29 genes were annotated to embryo implantation, 26 genes were categorized to placenta development, and 17 genes from this category were annotated to embryonic placenta development, whereas 9 genes were annotated to maternal placenta development (Fig. 7).

Additionally, molecular pathways were annotated from the Kyoto Encyclopedia of Genes and Genomes database (KEGG). Purine metabolism, biosynthesis of antibiotics, and pyrimidine metabolism were the most numerous, with 260, 219, and 120 sequences in the pathway, respectively (Online Resource 3).

### Single nucleotide variant prediction

**Fig. 8:**
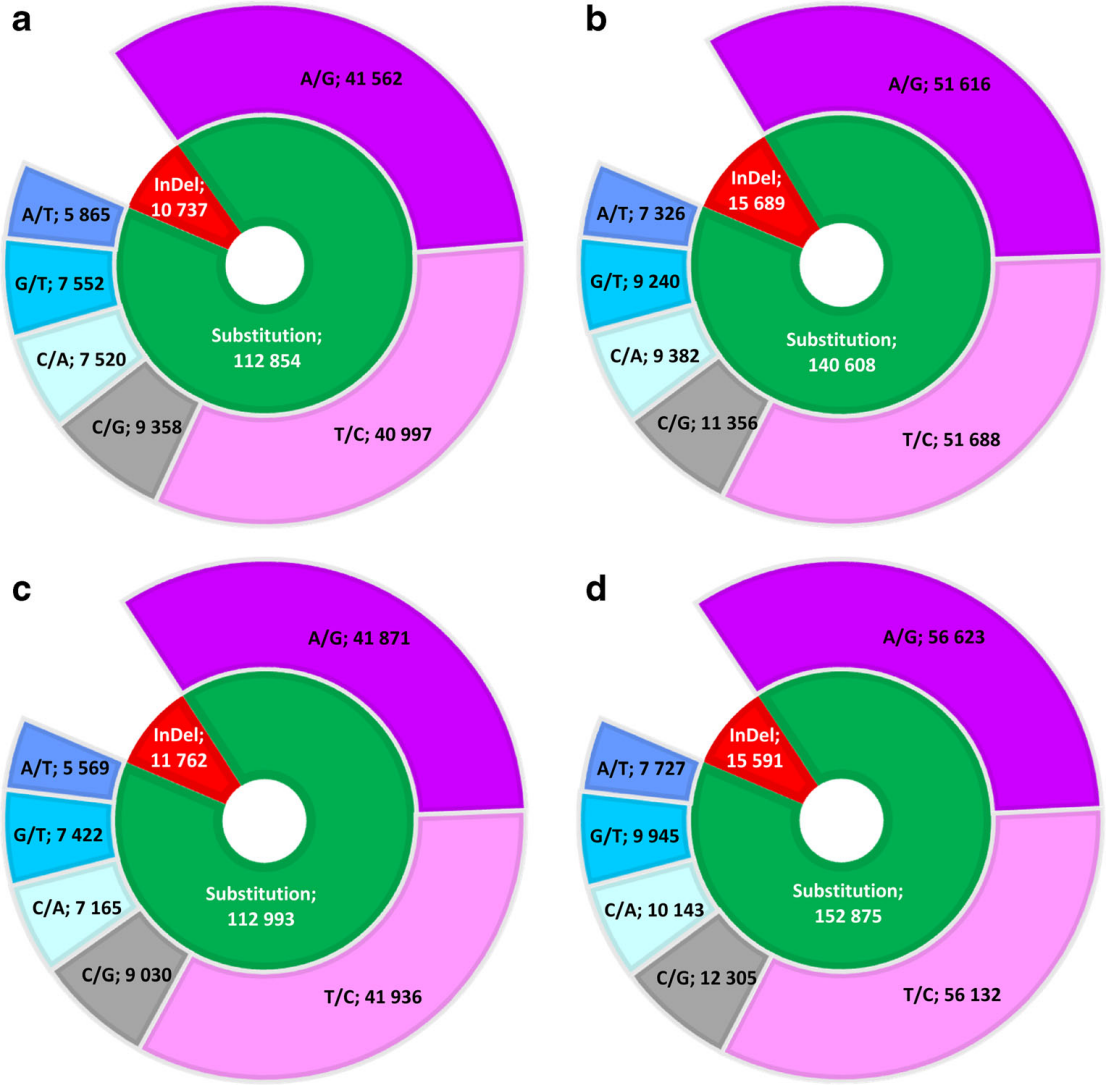
Summary of variants detection for each human placental sample (**a** Hs_p3, **b** Hs_p9, **c** Hs_p12, **d** Hs_p14). Numbers of single nucleotide variants (SNVs) and insertions–deletions (InDels) found for each sample are presented in the *inner circle*. In addition, transitions (A/G and C/T) and transversions (A/T, A/C, G/T, C/G) are presented in the *outer circle*

GATK and the Picard tool revealed the variant profile in each sample, and in total, 573,109 changes were predicted, 123,591, 156,297, 124,755, and 168,466 in sample Hs_p3, Hs_p9, Hs_p12, and Hs_p14, respectively (Fig. 8). In all samples, single nucleotide variants (SNVs) were more numerous (112,854–152,875) than InDel variations (10,737–15,689).

Variant (insertion, deletion, and substitution) detection was conducted for the contigs exclusively found for each GO term: embryo development, system development, embryo implantation, and embryonic and maternal placenta development (Table 4).

**Table 4:** SNVs/InDels distribution according to the five GO (Gene Ontology) categories in each placental sample (for definitions of samples see Table 1)

GO category	Sample			
	Hs_p3	Hs_p9	Hs_p12	Hs_p14
Placenta development GO: 0001890	222/34	256/29	279/36	262/35
Maternal placenta development GO: 0001893	75/11	100/18	97/25	145/20
Embryonic placenta development GO: 0001892	200/35	338/54	277/33	274/63
Embryo implantation GO: 0007566	422/49	482/62	481/52	518/64
Embryo development GO: 0009790	670/87	851/106	692/85	1,055/119

*Hs* human sample

Among all 1153 SNVs within the placenta development category, 1019 (88.38%) consisted in substitutions, while 67 (5.81%) were insertions as well as 67 (5.81%) were deletions. Meanwhile, 491 variants were detected in the maternal placenta development category, among which 417 (84.93%) were substitutions, 43 (8.76%) and 31 (6.31%) were insertions and deletions, respectively. In the embryonic placenta development category, 1189 (86.54%) were substitutions, 97 (7.06%) were insertions, and 88 (6.40%) were deletions, giving a total of 1374 variants. Within the 2130 variants predicted in embryo implantation, 1903 (89.34%), 122 (5.73%), and 105 (4.93%) were substitutions, insertions, and deletions, respectively. Among embryo development variants (3665), 3268 (89.17%) consisted in substitutions while 242 (6.60%) were insertions, and 155 (4.23%) were deletions. The full list of SNVs identified for each analyzed GO category is provided in Online Resource 4.

## Discussion

In the current study, we performed RNA-Seq to characterize the transcriptome profile of the human placenta term pregnancies ended by cesarean section. Our thorough analyses of placental transcriptome permitted the identification of transcripts’ profile, novel alternative junction events, and potential SNV within the entire placental transcriptome. Results obtained in our study complement current findings in the field of molecular profiling of pregnancy-depended transcriptome changes in various tissues (Adams et al. 2011; Sharp et al. 2016).

We obtained 119,560,140 total raw reads with 196–97 bp average read length from four placental term tissue, whereas Kim et al. (2012) generated paired-end data with 54 and 72 bp in read length and 23–33 million reads of each lane, for a total of 50–60 million paired-end reads per tissue for each of the placental tissues (amnion, chorion, and decidua). We obtained a high mapping rate with uniquely mapped reads in the range of 84.29–87.72% of reads aligned to the reference genome and only 1.53, 6.95, 7.44%, and 4.67% (for each of sample) of total unmapped reads, which indicates successful cDNA library construction and good sequencing quality of the human placental transcriptome. Using the uniquely mapped read pairs, Kim et al. (2012) estimated the expression levels of 22,523 protein-coding genes for single placental tissue, and approximately half of the genes are expressed with FPKM >1. Among total expressed TARs, our research revealed 14,785–16,202 protein-coding genes with levels of expression FPKM >1 for four placental tissues. Our data show that the largest number of genes was expressed below 1 FPKM (Fig. 3).

In our research, we calculated FPKM values for every transcript from each sample to generate an overview on placental transcriptome. The highest FPKM values were determined for high temperature requirement protease A1 (*HTRA1*), endothelial PAS domain protein 1 (*EPAS1*), and Pleckstrin homology-like domain, family a, member 2 (*PHLDA2*).

The physiological function of HTRA1 remains unclear. However, human HTRA1 has been linked with the pathogenesis of several abnormalities, such as osteoarthritis, rheumatoid arthritis, and even cancers (Hasan et al. 2015). Additionally, HTRA1 concentration increases in the maternal plasma of gestations complicated by preeclampsia with intrauterine growth restriction compared with control (D’Elia et al. 2012). *EPAS1* encodes a transcription factor involved in the response to fluctuating oxygen levels. There are conflicting reports concerning associations of *EPAS1* mutations and expression level with abnormalities during pregnancy. However, *EPAS1* is considered a candidate gene as a potential biomarker of the susceptibility, early detection, and/or individualized maternal–infant care in case of preeclampsia (Founds et al. 2011). PHLDA2 regulates fetal growth, placental development, and placental lactogen production (Tunster et al. 2013; Jensen et al. 2014; Janssen et al. 2016). Abnormally increased placental *PHLDA2* expression is associated with fetal growth restriction in human pregnancies and/or low birth weight (Jensen et al. 2014). Increased *PHLDA2* expression is also reported in cases of spontaneous miscarriage or fetal death (Doria et al. 2010). Moreover, increased placental *PHLDA2* expression correlates with reduced fetal movements (RFM) pregnancies. Because of its impact on pregnancy outcome, *PHLDA2* is postulated as a placental prenatal diagnostic biomarker identifying RFM pregnancies (Janssen et al. 2016). For our study, we selected patients with uncomplicated pregnancies, and thus we cannot exclude that high level of placental mRNA for *HTRA1* does not affect its serum level. However, we also cannot exclude that some undiagnosed or unnoticeable abnormality might have occurred in samples with high expression of *HTRA1*, *PHLDA2*, and *EPAS1*.

In our study, searching unannotated regions against the nr database revealed 134 transcripts with ORF longer than 300 bp composed of at least 2 exons and similarity less than 90%. In similar studies, 604, 1007, and 896 novel TARs in amnion, chorion, and decidua, respectively, not overlapping with the annotated transcripts from the NCBI RefSeq, UCSC, Ensembl, and Vega database were identified (Kim et al. 2012). Thus, taking into consideration the spatio-temporal changes of the transcriptome landscape, the differences between the obtained results and previous studies are inescapable.

Additionally, our studies uncovered 6497 splice junctions with high-quality coverage, and among this group 30 were verified as novel splice junctions.

Among the identified 30 novel splice junctions, we considered pregnancy-associated plasma protein A (*PAPPA*) and hemoglobin subunit alpha (*HBA*) the most interesting in the context of our research. PAPPA is a metalloprotease belonging to the metzincin superfamily of zinc peptidase (Leguy et al. 2014). It is an active homodimer (dPAPPA), circulating at very low levels in non-pregnant women and men, and regulates cell differentiation and proliferation by cleaving insulin-like growth factor binding proteins 4 and 5 (D’Elia et al. 2012; Coskun et al. 2013). During pregnancy, PAPPA is produced at high levels by the placenta and circulates as a heterotetrameric complex (htPAPPA). Decreased levels of this complex are associated with adverse pregnancy outcomes such as intrauterine growth restriction (IUGR), preterm delivery, miscarriage, preeclampsia, or fetal aneuploidy (Kirkegaard et al. 2010; Ranta et al. 2011). According to The World Health Organization, deletions of the α-globin genes are the most common genetic disorders in the world, and afflict 20% of the world’s population (Modell and Darlison 2008). The deletion of one or two α-globin genes may provide positive effects such as protection against malaria (Harteveld and Higgs 2010). However, the deletion of three genes causes thalassemia, which often results in significant anemia. Moreover, the deletion of all four α-globin genes results in fetal death and significant risk of maternal morbidity or mortality (Leung et al. 2008). The development of high-throughput RNA-seq and bioinformatic analyses enabled the detection of alternative splicing events (Wang et al. 2009). In general, genes contain multiple introns, with an average of 7.8–9.0 introns per gene in vertebrates. Previous RNA-seq analyses revealed that >80% of mammalian genes include introns, and >95% of transcripts in humans are alternatively spliced (Pan et al. 2008; Wang et al. 2008). Alternative splicing can generate various transcripts and thus multiple protein isoforms from a single gene. This mechanism allows the modulation of gene function, phenotype, and eventually cause diseases (Mourier and Jeffares 2003). Transcriptome and proteome diversity can be generated by exon skipping, alternative 5′ donor sites, and intron retention (Li et al. 2014). Regulation of alternative splicing (AS) events in multi-exon genes not only affects expression level or protein function, but can also lead to differences in AS between human individuals (Wang et al. 2008; Lu et al. 2012). Changes in coding sequence may lead to disturbances in functions, due to the presence or absence of specific protein domains. Alternative splicing events may also be present in promoter and UTR regions affecting expression level and isoform localization, respectively (An et al. 2008). Alternative splicing events regulate gene expression, which is essential for cell differentiation and organism development (Horiuchi and Aigaki 2006; Pan et al. 2008; Nilsen and Graveley 2010). It is mostly unknown how splice variants differ in terms of properties, but it is still incontestable that alternative splicing may increase the functional diversity in positive and negative manners (Polo-Parada et al. 2004; An et al. 2008). Alternative splicing as a universal regulatory mechanism has a significant impact on the tissue-specific transcriptome landscape (Wang et al. 2008). The example of the placental matador gene splice isoform that is responsible for trophoblast cell death in preeclampsia (Soleymanlou et al. 2005) shows how placental transcriptome may affect embryo development. Additionally, this underlines the importance of research aimed at characterizing placental transcriptome. We can assume that the novel placental transcripts identified in this study (such as PAPPA and HBA) may contribute to pregnancy course, but further functional analyses are required to state how they affect pregnancy outcome.

To gain more insight into placental functioning during uncomplicated pregnancy, we performed functional annotation analysis of placental transcripts that may be useful in understanding the functional significance of the placental transcripts, and in the future may constitute the answer to how they are implicated in placental biology and/or pregnancy disorders. In a similar study, differentially expressed placental enriched transcripts were mostly assigned to the following GO categories: multicellular organismal process, single-multicellular organism process, and response to stress (Hughes et al. 2015). We performed GO analyses for all placental transcripts with FPKM >1 to generate an overview of the placental landscape. In our analyses, transcripts annotated to biological processes were distributed in 16 subcategories. Over the represented category was the cellular process. However, most enriched GO terms (Hughes et al. 2015) were also represented in our results. In the general overview, it may be observed that placental expression is very diversified and numerous, and distinct GO categories were represented (Figs. 6 and 7). This is probably the effect of wide placental functions, including the transport of gases, nutrients and waste products, hormone production, and protection of the fetus from the maternal immune system (Kay et al. 2011).

Next-generation sequencing, including deep sequencing of the complementary RNA-seq, allows for global high-throughput analyses, leading to the identification of SNVs within known genes, novel transcripts, alternative splicing variants, trans-splicing events, and to adjusting predicted gene models (Wang et al. 2008, 2009). The obtained results regarding SNVs within genes associated with reproduction may constitute a basis for further genome-wide association (GWA) studies (Appels et al. 2013; Akpinar et al. 2017). The relationship between a specific genotype and a phenotype can be used to predict genes that may be correlated with observable traits in animals. GWA studies based on NGS results do not depend upon prior knowledge of the biological pathways, and this provides the opportunity to discover SNPs associated with phenotype (Liaoa and Leeb 2010). However, in the case of our results, it is possible to predict SNVs that may be involved in proper placental development or that induce pathology during pregnancy, which can seriously affect pregnancy outcome. In the light of recent research showing that ~20% of infertility has an unknown background (Iammarrone et al. 2003), our study is in line with the search for genetic markers that may be associated with idiopathic infertility.

In conclusion, we characterized the transcriptomic profile of placental tissue and identified novel transcripts expressed in term human placenta that may be involved in pregnancy course. These results may provide a basis for further analyses of the pathways essential for proper placental development and functioning that contribute to the pregnancy outcome. Overall, these results provide new resources for the identification of mechanisms regulating placental physiology during non-complicated pregnancy.

## Electronic supplementary material

Online Resource 1 InterproScan predicted signatures within Panther, Pfam and SMART databases (XLSX 381 kb)

Online Resource 2 Direct Gene Ontology (GO) categorization of placental genes to biological process, cellular component and molecular function (XLSX 1061 kb)

Online Resource 3 Placental genes classification to molecular pathways due to the Kyoto Encyclopedia of Genes and Genomes (KEGG) database (XLSX 43 kb)

Online Resource 4 SNVs identified in each sample for analyzed GO category (embryo development, embryo implantation, embryonic placenta development, maternal placenta development and placenta development) (XLSX 402 kb)
